# Pretransplant Malnutrition Risk Components are Associated With Adverse Outcomes After Simultaneous Pancreas and Kidney and Solitary Pancreas Transplantation

**DOI:** 10.1111/ctr.70607

**Published:** 2026-07-02

**Authors:** Katrina Kennedy, Jessa Engelken, Megan Rolfson, Angela Zhou, Mina L. Gibes, Najiyah Salwa, Didier Mandelbrot, Dixon Kaufman, Jon Odorico, Sandesh Parajuli

**Affiliations:** ^1^ Division of Transplantation, Department of Clinical Nutrition UW Health Hospital and Clinics Madison Wisconsin USA; ^2^ Division of Nephrology, Department of Medicine University of Wisconsin School of Medicine and Public Health Madison Wisconsin USA; ^3^ Division of Transplant Surgery, Department of Surgery University of Wisconsin School of Medicine and Public Health Madison Wisconsin USA

**Keywords:** malnutrition, outcomes, pancreas transplant

## Abstract

**Background:**

Malnutrition is common among patients with end‐organ disease. The impact of any criterion of malnutrition (weight loss, muscle depletion, fat depletion, inadequate oral intake, or reduced functionality) pretransplant among simultaneous pancreas‐kidney (SPK) or solitary pancreas transplant (SPT) recipients is understudied.

**Methods:**

In this single‐center study, we included all SPK or SPT recipients who had pretransplant malnutrition assessed and were transplanted between 01/2016 and 04/2024. Recipients were categorized as at malnutrition risk (M+ve) if any one of the components of malnutrition was present and were compared with those without any component (M‐ve). Outcomes of interest were length of stay, kidney delayed graft function (DGF), early readmission, cardiovascular events, acute rejection, uncensored graft failure, death‐censored graft failure (DCGF), and death.

**Results:**

A total of 234 SPK and 136 SPT recipients fulfilled the selection criteria. 44 (19%) of SPK recipients and 13 (10%) of SPT were MR+ve, with reduced functionality being the most common positive malnutrition component in both groups. Among SPK recipients, after adjustment for multiple variables, MR+ve was associated with early readmission (aOR: 1.79; 95% CI: 1.08–2.97; *p* = 0.02). Similarly, among SPT, MR+ve was associated with early readmission (aOR: 6.15; 95% CI: 2.43–15.5; *p* < 0.001); pancreas uncensored graft failure (aOR: 3.3; 95% CI: 1.14–9.58; *p* = 0.03) and pancreas DCGF (aOR: 4.45; 95% CI: 1.44–13.67; *p* = 0.009).

**Conclusion:**

The presence of any positive pretransplant malnutrition components is associated with worse posttransplant outcomes in pancreas transplant recipients. These findings help support informed decisions about transplant candidacy and nutrition optimization.

**Abbreviations**
AMC: Adult Malnutrition Criteria
AMR: Antibody‐mediated rejection
AND: Academy of Nutrition and Dietetics
ASPEN: American Society of Parenteral and Enteral Nutrition
BW: Body weight
CKD: Chronic kidney disease
DCGF: Death‐censored graft failure
EER: Estimated energy requirements
ESKD: End‐stage kidney disease
KDPI: kidney donor profile index
M+ve: Positive for malnutrition component
M‐ve: Negative for malnutrition component
NFPE: Nutrition focused physical exam
PEM: Protein‐energy malnutrition
RDN: Registered dietitian nutritionist
SPK: Simultaneous kidney pancreas
SPT: Solitary pancreas transplant
TCMR: T‐cell mediated rejection

## 1 Introduction

The term ‘malnutrition’ has no universally accepted definition. However, it is commonly described as a deficiency or imbalance of nutrients, resulting in a measurable adverse effect not only on body composition but also on function and clinical outcomes [1]. Under‐nutrition is the most common form of malnutrition, which is denoted by insufficient energy and nutrient intake to maintain health [2]. As depletion is frequently a combination of protein and energy shortage, the term protein‐energy malnutrition (PEM) became widely recognized in this field [3]. However, diagnostic criteria for PEM were never standardized and evolved with time [4]. Initially, various biochemical, anthropometric, immunological, and clinical measurements were used, including body weights, mid‐arm muscle circumference, triceps skinfold thickness, along with the use of serum albumin, transferrin, or total‐iron binding capacity as components of nutritional assessment [4]. Later, serum albumin and transferrin became highly utilized markers of malnutrition among hospitalized patients, but these markers were later mostly considered as markers of inflammation [4, 5]. In 2012, the Academy of Nutrition and Dietetics (AND), and the American Society for Parenteral and Enteral Nutrition (ASPEN) collaborated to develop a standardized set of diagnostic characteristics called the Adult Malnutrition Criteria (AMC for the purpose of this paper) [6]. The AMC is now embedded in Registered Dietitian Nutritionist (RDN) training and is highly utilized in clinical practice. The six characteristics in the AMC include inadequate energy intake, weight loss, loss of subcutaneous fat, localized or generalized fluid accumulation, loss of muscle mass, and diminished functional status as measured by hand grip strength [6]. The presence of 2 of these 6 criteria meets the definition of malnutrition [6].

Malnutrition is highly prevalent among patients with chronic kidney disease (CKD) and end‐stage kidney disease (ESKD) [7]. One meta‐analysis from 2018 estimated the global prevalence of malnutrition for patients on dialysis to be 28%–52% and 11%–54% in patients with CKD stages 3–5 [8, 9]. The prevalence of malnutrition among patients with diabetes is also high. In one meta‐analysis of 46 studies, with more than 18,000 patients, the overall prevalence of malnutrition among patients with diabetes was 33%, and 44% were at risk for malnutrition [10]. Most potential pancreas transplant recipients would have both CKD and diabetes for many years, and patients with combinations of these comorbidities would likely have an even greater prevalence of malnutrition [11]. The causes of malnutrition among patients with diabetes and CKD are multifactorial, including decreased nutrient intake associated with uremic symptoms, various dietary restrictions, and a heightened catabolic state [12].

The effect of pretransplant malnutrition or any positive component of pretransplant malnutrition on posttransplant outcomes of pancreas transplant recipients, either simultaneous pancreas and kidney (SPK) or solitary pancreas transplant (SPT), who were otherwise deemed suitable for pancreas transplant, is understudied. We hypothesized that patients possessing pretransplant malnutrition or any positive component of malnutrition would have inferior posttransplant outcomes.

## 2 Materials and Methods

This was a single‐center retrospective cohort study of pancreas transplant recipients (both SPK and SPT) who were transplanted between 01/01/2016 and 04/30/2024 and who had malnutrition status assessed pretransplant based on the AND/ASPEN AMC criteria. Outcomes were analyzed as of 01/2025. Of the six components (insufficient energy intake, weight loss, loss of subcutaneous fat, localized or generalized fluid accumulation, loss of muscle mass, and diminished functional status as measured by hand grip strength), only five components (all except for the fluid accumulation) were assessed. Although fluid accumulation is an important component for the diagnosis of malnutrition, this diagnosis is based largely on clinical criteria and has extremely poor diagnostic accuracy among patients with CKD and ESKD [13]. For this reason, fluid accumulation was not assessed. To overcome the potential impact of the dialysis access on grip strength on the same side, grip strengths were measured in the dominant hand.

Although we initially planned to assess outcomes based on malnutrition defined with at least two positive criteria, due to an extremely small sample size for patients with two components of malnutrition, and the fact that five rather than six components were assessed (fluid accumulation excluded), recipients were categorized based on having any of the five assessed components for malnutrition (M+ve) and were compared to those without any positive component (M‐ve). SPK and SPT recipient outcomes were analyzed separately. Multiorgan transplant recipients with a pancreas transplant, except for SPK, were not included. Outcomes of interest included length of stay (LOS) after index transplant, kidney delayed graft function (DGF) among SPK recipients only, early readmission within 30 days of discharge after index transplant, any cardiovascular events requiring hospitalization during the study period, biopsy‐proven pancreas rejection, pancreas death censored graft failure (DCGF), biopsy proven kidney rejection (among SPK only), kidney DCGF (among SPK only), and death with at least one functional graft.

This study was approved by the University of Wisconsin Health Sciences Institutional Review Board (IRB protocol number: 2014–1072) and adhered to the Declaration of Helsinki. The clinical and research activities reported were consistent with the Principles of the Declaration of Istanbul on Organ Trafficking and Transplant Tourism. Due to the nature of this study, informed consent specific to this research was not obtained from patients.

### 2.1 Malnutrition Diagnosis

Malnutrition was assessed in all potential pancreas transplant recipients since 2016. Malnutrition assessments were performed by the transplant dietitian during their pretransplant evaluation in the ambulatory clinic as described before [14]. The Registered Dietitian Nutritionists (RDN) completed a thorough assessment, including weight and diet history, along with the Nutrition Focused Physical Exam (NFPE) to assess muscle and fat depletion and evaluation of reduced functionality, as measured by hand grip strength [6]. During the initial wave of the COVID‐19 pandemic, to decrease patient contact, the reduced functionality component, typically assessed by grip strength, was not formally obtained in some of the low‐risk patients (*n* = 70 SPK, *n* = 0 SPT); and assigned not to have reduced functionality based on the clinical judgement and alternative assessment was performed and is described below. Patients were assigned 0 or 1 for each component based on the negative or positive findings.

The five malnutrition components differ slightly depending on whether the findings are classified as moderate or severe. In the context of chronic malnutrition (present for >3 months), weight loss aligning with moderate malnutrition includes 5% body weight (BW) loss x 1 mo, 7.5% BW loss x 3 mo, 10% BW loss x 6 mo, or 20% BW loss x 12 mo. Inadequate energy intake, estimated with a detailed diet recall, associated with moderate malnutrition is <75% of estimated energy intakes (EER) for >/ = 1 mo. Moderate and severe muscle and fat depletion are identified with a hands‐on nutrition‐focused physical exam following rigorous training by the RDN on identifying muscle and fat losses. Per AND/ASPEN AMC criteria, reduced functionality cannot be used in a moderate malnutrition diagnosis. Weight loss aligning severe malnutrition includes >5% BW loss x 1 mo, >7.5% x 3 mo, >10% x 6 mo, or >20% x 12 mo. Inadequate energy intake associated with severe malnutrition is </ = 75% of EER for >/ = 1 mo. In times when hand grip strength for functional assessment could not be obtained, questions about ability to do physical activity, including ADL/iADLs and a timed sit to stand test were implemented over video. Fluid accumulation is typically not used due to it's poorer diagnostic value given the prevalence of fluid shifts in many chronic conditions.

Malnutrition assessment and the severity of findings were discussed during pretransplant selection meetings and considered as one of the factors in deciding transplant candidacy. Patients on the waitlist were re‐evaluated every 1 to 2 years, including the malnutrition assessment. Patients with malnutrition or those at high risk for developing malnutrition were assessed at least yearly. Among patients with multiple malnutrition assessments, the malnutrition assessment closest to the transplant admission was used for analysis.

### 2.2 Immunosuppressive Protocols

Our center‐specific induction immunosuppression therapy was consistent throughout the study period; either a depleting agent (alemtuzumab or anti‐thymocyte globulin) or a nondepleting agent (basiliximab) was utilized. Triple immunosuppression with tacrolimus, mycophenolic acid, and a prednisone taper was standard for all recipients. A few patients had early steroid withdrawal based on the immunological risk and patient request, as previously described [15].

### 2.3 Biopsy and Rejection Protocols

The two most common indications for kidney biopsy were an unexplained rise in creatinine and proteinuria, or a human leukocyte antigen (HLA) donor‐specific antibody (DSA) based protocol biopsy, as described before [16]. Similarly, the common indications for pancreas biopsy were an unexplained rise in pancreatic enzymes, development of denovo DSA, and unexplained hyperglycemia. If possible, we attempt to perform both graft biopsies in the setting of dual graft dysfunction among SPK recipients [17].

Similarly, the management of rejection was based on the severity and proximity of the transplant to the diagnosis of rejection as described before [16, 17]. Briefly, kidney T cell‐mediated rejection (TCMR) was treated with steroid pulse with or without anti‐thymocyte globulin. Antibody‐mediated rejection (AMR) was treated with steroid pulse, intravenous immunoglobulin (IVIG), with or without rituximab, with or without plasmapheresis. Treatment of pancreas rejection was based on the type and severity of rejection and was graded by the Banff criteria. TCMR was treated with IV steroid pulse with or without anti‐thymoglobulin 6 to 12 mg/kg in 4 to 10 divided doses, while mixed rejection was treated with steroids, anti‐thymoglobulin, IVIG, and plasmapheresis. Early AMR was treated with steroids, IVIG, and plasmapheresis.

### 2.4 DGF Management

Patients with DGF are followed as outpatients in a dedicated DGF clinic, as previously described [18]. They were either discharged to their homes or a nearby hotel with a support person and were required to attend a clinic visit within 1–3 days post‐discharge. Dialysis was scheduled as needed on the same day in our inpatient dialysis unit.

## 3 Statistical Analysis

Continuous data were compared using Student's *t*‐test or the Wilcoxon rank‐sum test, or Kruskal–Wallis test as appropriate, while categorical data were analyzed using Fisher's exact test or chi‐square test (*χ* ^2^). *p* values ≤0.05 were considered statistically significant. Factors associated with outcomes of interest were computed using univariate and multivariable analyses. However, due to the limited incidence of outcomes of interest, multivariable models included only some of the clinically relevant factors including recipients' age, sex, BMI, type of diabetes, previous transplant, preemptive transplant, calculated panel reactive antibody (cPRA)> 20%, use of depleting induction agent, kidney donor profile index (KDPI) along with some donor factors including age, sex and BMI. Some of the significant outcomes were also presented as a Kaplan–Meier survival curve. All analyses were performed using the MedCalc Statistical Software version 16.4.3 (MedCalc Software, Ostend, Belgium; https://www.medcalc.org; 2016).

## 4 Results

### 4.1 Simultaneous Pancreas and Kidney Recipients

A total of 234 SPK recipients were included. 44 (19%) had at least one positive component of malnutrition (M+ve), and the remaining 190 (81%) did not have any positive component (M‐ve). Most of the baseline characteristics were similar between the groups (Table 1), except the mean LOS was significantly longer in the M+ve group, at 11.9 ± 17.1 days compared to the 9.9 ± 6.2 days (*p* < 0.001) in the M‐ve group. Also, there were differences in the utilization of immunosuppression between the groups.

**TABLE 1:** Baseline clinical characteristics of SPK.

Variables	All	At least one positive component of malnourishment	No‐positive component	*p*
Total number of recipients	234	44	190	
Recipient: Male (%)	158 (68)	31 (71)	13 (30)	0.65
Recipient: White (%)	171 (73)	32 (73)	12 (27)	0.95
Recipient: age at transplant	46.9 ± 9.9	48.9 ± 11.0	46.5 ± 9.6	0.21
Type of Diabetes				0.12
Diabetes I	136 (58)	21 (48)	115 (61)	
Diabetes II/Other/unknown	98 (42)	23 (52)	75 (40)	
Pre‐preemptive transplant (%)	41 (18)	6 (14)	35 (18)	0.45
Previous transplant recipients (%)	9 (4)	0	9 (5)	0.14
Recipients BMI (Kg/m2)	27.0 ± 3.6	26.7 ± 4.3	27.1 ± 3.4	0.46
Mean kidney donor profile index (%)	24.2 ± 18.0	21.0 ± 13.9	25.0 ± 18.8	0.18
Mean HLA mismatch of 6	4.6 ± 1.2	4.7 ± 1.0	4.5 ± 1.2	0.20
cPRA > 20% (%)	28 (12)	4 (9)	24 (13)	0.52
Kidney cold ischemia time (hrs)	14.2 ± 4.0	13.3 ± 3.6	14.4 ± 4.1	0.11
Pancreas cold ischemia time (hrs)	12.1 ± 3.9	11.3 ± 3.4	12.3 ± 4.0	0.13
Induction agent (%)				0.03
Basiliximab	17 (7)	0	17 (9)	
Anti‐thymocyte globulin	105 (45)	26 (59)	79 (42)	
Alemtuzumab	112 (48)	18 (41)	94 (49)	
Donor male (%)	153 (65)	32 (73)	121 (64)	0.26
reDonor white (%)	192 (82)	35 (80)	157 (83)	0.63
Donor age (yrs)	25.1 ± 11.2	26.6 ± 10.1	24.8 ± 11.4	0.33
Donors BMI (Kg/m2)	22.9 ± 3.8	23.7 ± 3.5	22.8 ± 3.9	0.12
Donor cause of death: Anoxia (%)	107 (46)	19 (43)	88 (46)	0.71
Early steroid withdrawal (%)	29 (12.4)	2 (5)	27 (14)	0.08
Mean posttransplant hospital stay (days)	10.3 ± 9.2	11.9 ± 17.1	9.9 ± 6.2	<0.001
Kidney DGF (%)	36 (15)	5 (11)	31 (16)	0.41

The mean interval from malnutrition assessment to transplant was 6.7 ± 4.3 months (Table 2). A total of 34 (15%) patients had at least one positive component, 6 had two positive components, 3 had three positive components, and 1 had all five positive components. The most common positive component was reduced functionality in 36 (15%) of the recipients. The mean LOS among the entire cohort was 10.3 ± 9.2 days. Having any positive component or positive for reduced functionality was not associated with prolonged or shortened LOS (Table S1).

**TABLE 2:** Malnutrition assessment of SPK.

Variables	
Mean interval from malnutrition assessment to transplant (mo)	6.7 ± 4.3
Any at least one component of malnutrition positive (%)	
Yes	44 (19)
No. of recipients with (%)	
One component positive	34 (15)
Two positive components	6 (3)
Three positive components	3 (1)
Four positive components	0
All five components positive	1
Weight loss	
0^a^	230 (98)
1^b^	4 (2)
Muscle depletion	
0	227 (97)
1	7 (3)
Fat depletion	
0	230 (98)
1	4 (2)
Poor oral intake	
0	225 (96)
1	9 (4)
Reduced functionality	
0	198 (85)
1	36 (15)

^a^ 0 indicates not present.

^b^ 1 indicates present.

The proportions of SPK recipients with other outcomes of interest, including kidney DGF, early readmission, cardiovascular events, pancreas rejection, pancreas DCGF, kidney rejection, kidney DCGF, or death, were also not significantly different between the groups (Table S2). A total of 105 recipients had an early readmission, with the most common indication for early readmission being gastrointestinal symptoms. In regression analysis, the only outcome of interest associated with M+ve was an increased risk for readmission in univariate analysis (OR: 2.45; 95% CI: 1.49–3.98; *p* < 0.001). This was further confirmed in multivariable analysis (OR: 1.79; 95% CI: 1.08–2.97; *p* = 0.02) (Table 3). Only one recipient had all 5 positive components of malnutrition, and this recipient did well without any negative outcomes of interest, even after more than 55 months since transplant.

**TABLE 3:** Association between malnutrition at least one positive component and clinical outcomes among SPK recipients.

	Malnutrition OR (unadjusted)	OR (adjusted)
Kidney DGF	1.02 (0.39–2.67; *p* = 0.96)	0.79 (0.29–2.17; *p* = 0.65)
Early readmission	**2.43 (1.49–3.98; *p* <0.001)**	**1.79 (1.08–2.97; *p* = 0.02)**
Cardiovascular events	0.84 (0.19–3.75; *p* = 0.82)	0.56 (0.12–2.67; *p* = 0.47)
Pancreas acute rejection	1.44 (0.48–4.35; *p* = 0.52)	1.11 (0.32–3.85; *p* = 0.87)
Pancreas death censored graft failure	1.10 (0.32–3.81; *p* = 0.88)	1.06 (0.29–3.88; *p* = 0.93)
Kidney acute rejection	1.15 (0.39–3.42; *p* = 0.79)	1.03 (0.33–3.21; *p* = 0.96)
Kidney death censored graft failure	1.24 (0.35–4.38; *p* = 0.73)	0.46 (0.09–2.28; *p* = 0.34)
Death with at least one functional graft	1.34 (0.44–4.05; *p* = 0.60)	1.32 (0.38–4.47; *p* = 0.66)

Adjusted for: Recipient's age, sex, BMI, type of diabetes, KDPI, depleting induction, preemptive transplant, previous transplant, cPRA >20%, donor's age, donor's sex, donor's BMI.

Among the 44 recipients in the M+ve group, the mean BMI was 26.7 ± 4.3 kg/m^2^, the mean HbA1c was 8.0 ± 1.8%, and the mean pretransplant serum albumin level was 3.4 ± 0.7 g/dL. Similarly, 33 recipients (75%) had Karnofsky performance scores >70%, indicating good functional capacity. Pretransplant neuropathy was present in 37 recipients (84%), and a similar proportion had retinopathy; additionally, 9 recipients (20%) had undergone partial lower‐extremity amputation. Posttransplant, only 1 recipient developed a surgical site infection, and 2 developed incisional hernias.

In a subgroup analysis, only 10 SPK recipients had ≥2 positive malnutrition components, meeting diagnostic criteria for malnutrition. None of these recipients experienced kidney DGF, cardiovascular events, or death with at least one functioning graft during follow‐up. In Cox regression analyses, malnutrition was not associated with an increased risk of hospital readmission (HR 2.09, 95% CI 0.91–4.81; *p* = 0.08), pancreas rejection (HR 1.27, 95% CI 0.17–9.48; *p* = 0.82), pancreas death‐censored graft failure (DCGF) (HR 1.50, 95% CI 0.20–11.29; *p* = 0.69), kidney rejection (HR 1.25, 95% CI 0.17–9.26; *p* = 0.83), or kidney DCGF (HR 3.21, 95% CI 0.73–13.97; *p* = 0.12).

### 4.2 SPT Recipients

A total of 136 SPT recipients were included. 14 were pancreas after kidney (PAK) transplant recipients, and the remaining were SPT recipients. 13 (10%) had at least one positive component of malnutrition (M+ve), and the remaining 123 (90%) did not have any positive components. Most of the baseline characteristics were similar between the groups (Table 4), except the proportion of white recipients was lower in the M+ve group (85% vs 97%, *p* = 0.04), along with the recipients' BMI being lower in M+ve group (24.7 ± 2.6 vs 27.5 ± 4.1, *p* = 0.01) compared to the M‐ve.

**TABLE 4:** Baseline clinical characteristics among SPT recipients.

Variables	All	Any component of malnourishment positive	Not‐malnourished	*p*
Total number of recipients	136	13	123	
Recipient: Male (%)	61 (45)	7 (54)	54 (44)	0.49
Recipient: White (%)	130 (96)	11 (85)	119 (97)	0.04
Recipient: age at transplant (yrs)	44.7 ± 11.6	47.2 ± 10.1	44.4 ± 11.7	0.41
Type of Diabetes				0.81
Diabetes I	123 (90)	12 (92)	111 (90)	
Diabetes II/Other/unknown	13 (10)	1 (8)	12 (10)	
Previous transplant recipients (%)	30 (22)	5 (39)	25 (20)	0.14
Recipients BMI (Kg/m2)	27.2 ± 4.1	24.7 ± 2.6	27.5 ± 4.1	0.01
Mean HLA mismatch of 6	4.7 ± 1.1	4.4 ± 1.4	4.7 ± 1.0	0.12
cPRA > 20% (%)	37 (27)	3 (23)	34 (28)	0.73
Pancreas cold ischemia time (hrs)	13.3 ± 3.8	13.5 ± 4.3	13.3 ± 3.8	0.48
Induction agent (%)				0.19
Basiliximab	2 (2)	0	2 (2)	
Anti‐thymocyte globulin	62 (46)	3 (23)	59 (48)	
Alemtuzumab	72 (53)	10 (77)	62 (50)	
Donor male (%)	86 (63)	5 (39)	81 (66)	0.05
Donor white (%)	95 (70)	11 (85)	84 (68)	0.22
Donor age (yrs)	25.9 ± 10.6	30.6 ± 12.9	25.3 ± 10.3	0.22
Donors BMI (Kg/m2)	23.3 ± 3.8	23.2 ± 4.1	23.3 ± 3.8	0.65
Donor cause of death: Anoxia (%)	58 (43)	4 (31)	54 (44)	0.36
Early steroid withdrawal (%)	26 (19)	2 (15)	24 (20)	0.72
Mean posttransplant hospital stay (days)	7.2 ± 3.1	7.8 ± 4.1	7.2 ± 3.0	0.10

The mean interval from malnutrition assessment to transplant was 4.3 ± 3.1 months (Table 5). 9 (7%) had at least one positive component, 1 had two positive components, 2 had four positive components, and 1 had all five positive components. The most common positive component was reduced functionality in 9 (7%) of the recipients. The mean LOS among the entire cohort was 7.2 ± 3.1 days. Having any positive component or positive for reduced functionality was not associated with prolonged or shortened LOS (Table S3). The proportions of SPT recipients with other outcomes of interest, including early readmission, cardiovascular events, pancreas rejection, pancreas DCGF, and death, were also not significantly different between the groups (Table S4). A total of 53 recipients had an early readmission, with the most common indication for early admission being gastrointestinal symptoms.

**TABLE 5:** Malnutrition assessment (SPT recipients).

Variables	
At least one component of malnutrition positive (%)	
Yes	13 (10)
No. of recipients with (%)	
One positive component	9 (7)
Two positive components	1(1)
Three positive components	0
Four positive components	2 (1)
All five components positive	1(1)
Weight loss	
0^a^	131 (96)
1^b^	5 (4)
Muscle depletion	
0	132 (97)
1	4 (3)
Fat depletion	
0	132 (97)
1	4 (3)
Poor oral intake	
0	134 (99)
1	2 (2)
Reduced functionality	
0	127 (93)
1	9 (7)

^a^ 0 indicates not present.

^b^ 1 indicates present.

**FIGURE 1:**
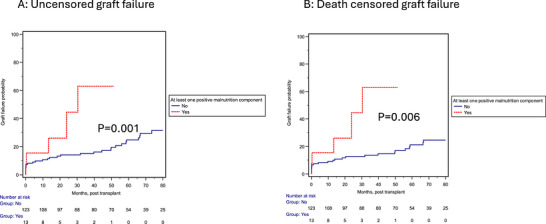
Increased risk of uncensored graft failure (Figure 1A, *p* = 0.001) and DCGF (Figure 1B, *p* = 0.006) among SPT recipients with any positive component of malnutrition.

In regression analysis, M+ve were associated with an increased risk for readmission in univariate analysis (OR: 5.7; 95% CI: 2.39–13.71; *p* < 0.001). This was further confirmed in multivariable analysis (OR: 6.15; 95% CI: 2.43–15.5; *p* < 0.001) (Table 6A). Similarly, M+ve was associated with increased risk for pancreas uncensored graft failure in univariate analysis (OR: 3.18; 95% CI: 1.18–8.54; *p* = 0.02) and multivariable analysis (OR: 3.31; 95 % CI: 1.14–9.58; *p* = 0.03), along with pancreas DCGF in univariate (OR: 3.67; 95% CI: 1.34–10.04; *p* = 0.01) and multivariable analysis (OR: 4.45; 95 % CI: 1.44–13.67; *p* = 0.009). Also, increased risk of pancreas uncensored and DCGF is presented in the unadjusted Kaplan‐Meier survival curve (Figure 1).

**TABLE 6:** Association between malnutrition with at least one component positive, and clinical outcomes among SPT recipients.

	Malnutrition OR (unadjusted)	OR (adjusted)
**A: All SPT recipients**		
Early readmission	**5.7 (2.39–13.71; *p* < 0.001)**	**6.15 (2.43, 15.5; *p* < 0.001)**
Cardiovascular events	5.9 (0.53–66.0; *p* = 0.15)	1.99 (0.13–31.6; *p* = 0.62)
Pancreas acute rejection	3.52 (0.77–16.0; *p* = 0.10)	4.20 (0.88–20.1; *p* = 0.07)
Pancreas uncensored graft failure	**3.18 (1.18–8.54; *p* = 0.02)**	**3.31 (1.14–9.58; *p* = 0.03)**
Pancreas death censored graft failure	**3.67 (1.34–10.04; *p* = 0.01)**	**4.45 (1.44–13.67; *p* = 0.009)**
Death with functioning graft	NA	NA
**B: PTA recipients only**		
Early readmission	1.79 (0.71–4.52; *p* = 0.22)	1.92 (0.69–5.31; *p* = 0.21)
Cardiovascular events	1.01 (0.98–1.04; *p* = 0.52)	1.98 (0.12–32.3; *p* = 0.63)
Pancreas acute rejection	1.01 (0.99–1.02; *p* = 0.15)	**5.30 (1.09–25.65; *p* = 0.03)**
Pancreas uncensored graft failure	**0.98 (0.97–0.99; *p* = 0.003)**	3.09 (0.87–11.0; *p* = 0.08)
Pancreas death censored graft failure	**0.98 (0.96–0.99; *p* = 0.002)**	**4.47 (1.18–16.87; *p* = 0.03)**
Death with functioning graft	NA	NA

Adjusted for: Recipient's age, sex, race, BMI, previous transplant, donor's age, donor's sex, donor's BMI.

Only one recipient had all 5 positive components of malnutrition, and that recipient had early graft failure within 2 weeks due to graft thrombosis.

Among the 13 recipients in the M+ve group, the mean BMI was 24.7 ± 2.6 kg/m^2^, the mean HbA1c was 8.3 ± 1.1%, and the mean pretransplant serum albumin level was 3.8 ± 0.4 g/dL. Similarly, 11 recipients (85%) had Karnofsky performance scores >70%. Pretransplant neuropathy was present in 9 recipients (69%), and a similar proportion had retinopathy; additionally, 1 recipient (8%) had undergone partial lower‐extremity amputation. Posttransplant, none developed a surgical site infection, and 2 (16%) developed incisional hernias.

When limiting to 122 pancreas transplant alone (PTA) recipients (by excluding 14 PAK recipients), only 9 (7%) had at least one positive component of malnutrition (M+ve), and the remaining 113 (93%) did not have any positive components. In regression analysis (Table 6B), M+ve were associated with an increased risk for pancreas acute rejection (OR: 5.30; 95 % CI: 1.09–25.65; *p* = 0.03), and pancreas DCGF (OR: 4.47; 95 % CI: 1.18–16.87; *p* = 0.03).

To further evaluate the association between malnutrition and posttransplant outcomes, we performed a subgroup analysis among SPT recipients meeting diagnostic criteria for malnutrition (≥2 positive malnutrition components). Only four recipients met these criteria. None experienced cardiovascular events or death with a functioning graft during follow‐up. In Cox regression analyses, malnutrition was not significantly associated with hospital readmission (HR 4.42, 95% CI 0.99–18.67; *p* = 0.06) or pancreas rejection (HR 4.50, 95% CI 0.56–34.49; *p* = 0.15). However, malnutrition was associated with a significantly increased risk of pancreas uncensored graft failure (HR 6.04, 95% CI 1.81–20.20; *p* = 0.03) and pancreas DCGF (HR 6.87, 95% CI 2.04–23.19; *p* = 0.002)

## 5 Discussion

In this large cohort of 234 SPK and 136 SPT recipients (a total of 370 pancreas transplant recipients), we report the prevalence of positive components of malnutrition to be more than 17%, with reduced functional status measured by hand grip strength being the most common. Likely due to stringent selection criteria to receive a pancreas transplant and strong consideration of nutrition risks, the prevalence of malnutrition components was not that high among these selected patients who were deemed suitable to receive a pancreas transplant and had undergone extensive workup before transplant with yearly nutrition follow up. Still, having any positive component of malnutrition was associated with detrimental outcomes, including early readmission in both SPK and SPT recipients and pancreas uncensored and pancreas DCGF among SPT (when PAK were also included). All of these findings highlight the importance of the assessment of malnutrition components at pretransplant evaluations to help providers make informed committee decisions about candidacy.

Malnutrition is a common and usually under‐recognized and untreated medical problem in clinical settings, usually among those with chronic medical conditions [19]. Even going back to the 1930's, surgeons noticed that underweight patients or those who were starved for a longer period had higher postoperative complications and mortality [19]. Now, it is well known that even in the general population (non‐transplant), malnutrition is associated with various adverse outcomes, including increased morbidity, mortality, increased risk for infection, poor wound healing, significantly longer LOS, increased healthcare utilization, decreased function, and poor quality of life [8]. Also, patients with insulin‐dependent diabetes are at increased risk of perioperative morbidities and complications. In one large cohort of 211,436 patients, of which 7.1% had insulin‐dependent diabetes and 9.8% with diabetes managed on oral medication, a total of 177,430 general surgeries and 34,006 vascular surgeries were performed [20]. Comparing the outcomes among patients with diabetes vs without diabetes, the authors report no significant difference in the rate of mortality. However, major morbidities that encompass wound infections, respiratory issues, urinary tract infections, central nervous system, and cardiac issues were significantly higher among patients with diabetes, and particularly among those with insulin dependent diabetes (11% higher comparing between patients with diabetes vs no diabetes and 20 and 24% higher among patients with diabetes on insulin vs oral agents undergoing for general surgeries and vascular surgeries respectively) [20]. Similarly, patients with CKD are at increased risk for post‐surgical mortality and major complications [21]. To summarize, patients with diabetes and CKD have a higher prevalence of malnutrition, and these patients are at an increased risk of perioperative morbidity and mortality. With this potential, pancreas transplant recipients are at risk of having all of these complications.

Malnutrition is highly prevalent in various other potential solid organ recipients awaiting transplant, and depends on the types of end organs, and duration of the disease, ranging from 10% to 90% [8]. In liver transplant candidates with cirrhosis, sarcopenia is associated with mortality on the waiting list, especially for those with lower Model for End‐Stage Liver Disease (MELD) scores. Modified MELD scores have been created to include sarcopenia scores, and when present, sarcopenia adds 10 points to the MELD score, demonstrating the relevance of muscle wasting as a prognostic factor [22]. Similarly, among heart and lung transplant candidates, there has not been much study based on AMC criteria for malnutrition; however, some of the variables included in those criteria were studied and found to have detrimental outcomes [8].

In one recent study from our group, with 367 kidney transplant recipients, we reported 10% were malnourished on pretransplant evaluation [14]. From the same study, recipients who were malnourished pretransplant were at significantly higher risk for DGF and early readmission. Our study also analyzed the individual components of malnutrition and found that muscle depletion was the most common positive component and was associated with poor outcomes [14]. In contrast, among SPK and SPT recipients, the reduced functionality component was highly prevalent. Of note, malnutrition assessments in the previous study among kidney‐only recipients and pancreas recipients in the current study were performed by the same group of RDNs, reducing interobserver variability. These results support the findings that patients with diabetes are at increased risk for reduced functionality, which could be due to multiple factors including poor glycemic control, hormonal imbalances, cognitive decline, polypharmacy, chronic underlying inflammation, and many more [23]. Interventions focusing on improving functionality and strength among patients with diabetes may have positive outcomes overall, including post‐pancreas transplant.

There is some overlap between frailty and malnutrition criteria; however, the two are not synonymous [24]. Jeejeebhoy et al. suggest that the use of malnutrition and frailty tools in combination may be valuable [25]. The apparent overlap suggests that a minimum set of indicators should be further defined and researched to determine their utility in terms of response to intervention, predicting adverse outcomes, and still be feasible in the fast‐paced clinical setting [24]. To answer some of these questions, in the past, our group has reported outcomes after analyzing individual components of frailty among pancreas transplant recipients (both SPK and SPT) [26]. In that study, we found among SPK recipients, slow walk time was associated with increased risk for mortality, while in SPT, unintentional weight loss and low grip strength were associated with poor outcomes [26]. Combining these two studies, it seems that low grip strength (which is also included in malnutrition assessment as reduced functionality), is the most important factor predicting poor posttransplant outcomes and should be included in the pretransplant assessment at a minimum.

This study has the expected limitations of a single‐center observational study, reflecting our specific population and clinical approach. Our findings are reflective of the practices at our center, and this should be factored into the interpretation. Due to the limited sample size of patients with a positive malnutrition diagnosis, it was not feasible to analyze outcomes based on the diagnosis of malnutrition itself. Therefore, we analyzed outcomes based on the presence of any single positive component of the malnutrition assessment. Outcomes may have differed among recipients who fully met the criteria for malnutrition. Also, we were not able to present data about potential candidates with malnutrition who were deemed not suitable for transplant. However, this substantial data set with more granular data provides useful information for estimating risks and outcomes. Also, to the best of our knowledge, this study is the largest of its kind, and we hope this study, along with our previously published research on frailty, will connect the intersection of frailty and malnutrition in the transplant population.

In conclusion, this study indicates the potential role of malnutrition assessments in the pretransplant period and their association with negative outcomes. We propose that each nutrition evaluation conducted by the RDN should include a comprehensive malnutrition assessment using the AND/ASPEN AMC criteria. This evaluation should include clear and effective nutrition interventions to optimize patients for transplantation and a clear plan for monitoring nutrition status in the pretransplant period, which may improve posttransplant outcomes. Potential nutrition interventions for optimization may include interventions to increase calorie and protein intake, improve glycemic control, and medical nutrition therapy for gastrointestinal conditions, such as gastroparesis and exocrine pancreatic insufficiency to improve nutrition reserve. These interventions may help improve posttransplant outcomes in these vulnerable patient populations. Further studies assessing longitudinal posttransplant malnutrition status, as well as pretransplant interventions aimed at improving malnutrition and related outcomes, are needed.

## Author Contribution

Kennedy: concept, data collection, design, manuscript preparation. Engelken: data collection, design, manuscript editing. Rolfson: data collection. Zhou: data collection, manuscript editing. Gibes: data collection, manuscript editing. Salwa: data collection, manuscript editing. Mandelbrot: editing. Kaufman: editing. Odorico: editing. Parajuli: concept, design, data analysis, manuscript preparation

## Funding

Dr. Mandelbrot has received research funds from the Virginia Lee Cook Foundation. The data that supports the findings of this study are available from the corresponding author, [SP], upon reasonable request.

## Disclosure

There are no conflicts of interest or sources of funding to disclose.

## Conflicts of Interest

The authors declare no conflicts of interest.

## Supporting information




**Supporting Information**: ctr70607‐supp‐0001‐Table S1.docx

**Supporting Information**: ctr70607‐supp‐0002‐Table S2.docx

**Supporting Information**: ctr70607‐supp‐0003‐Table S3.docx

**Supporting Information**: ctr70607‐supp‐0004‐Table S4.docx

## Data Availability

The data that support the findings of this study are available from the corresponding author upon reasonable request.
