# Supplementary material for: Pretransplant Malnutrition Risk Components are Associated With Adverse Outcomes After Simultaneous Pancreas and Kidney and Solitary Pancreas Transplantation
Source: Clin Transplant. 2026 Jul 2;40(7):e70607. doi: 10.1111/ctr.70607 (PMC13325689; doi:10.1111/ctr.70607)
Supplement: Supplementary file 1 — Supporting Information: ctr70607‐supp‐0001‐Table S1.docx [file CTR-40-e70607-s004.docx]

**Table S1: Length of Stay among SPK recipients**

|  | Correlation Coefficient (95% CI) |
| --- | --- |
|  |  |
| Any component positive | 0.08 (-0.05 to 0.21; p= 0.22) |
| Reduced functionality | 0.08 (-0.08 to 0.23; p= 0.33) |
