# Supplementary material for: Pretransplant Malnutrition Risk Components are Associated With Adverse Outcomes After Simultaneous Pancreas and Kidney and Solitary Pancreas Transplantation
Source: Clin Transplant. 2026 Jul 2;40(7):e70607. doi: 10.1111/ctr.70607 (PMC13325689; doi:10.1111/ctr.70607)
Supplement: Supplementary file 2 — Supporting Information: ctr70607‐supp‐0002‐Table S2.docx [file CTR-40-e70607-s002.docx]

**Table S2: Comparison of outcomes of interest among SPK recipients**

| Outcomes of interest | At least one positive component | None of the positive component | p |
| --- | --- | --- | --- |
| Kidney DGF | 5 (11) | 31 (16) | 0.41 |
| Early readmission | 23 (52) | 82 (43) | 0.27 |
| Cardiovascular events | 2 (5) | 22 (12) | 0.16 |
| Pancreas acute rejection | 4 (9) | 20 (11) | 0.78 |
| Pancreas death censored graft failure | 3 (7) | 19 (10) | 0.52 |
| Kidney acute rejection | 4 (9) | 29 (15) | 0.29 |
| Kidney death censored graft failure | 3 (7) | 16 (8) | 0.72 |
| Death with at least one functional graft | 4 (9) | 17 (9) | 0.98 |
