# Supplementary material for: Pretransplant Malnutrition Risk Components are Associated With Adverse Outcomes After Simultaneous Pancreas and Kidney and Solitary Pancreas Transplantation
Source: Clin Transplant. 2026 Jul 2;40(7):e70607. doi: 10.1111/ctr.70607 (PMC13325689; doi:10.1111/ctr.70607)
Supplement: Supplementary file 3 — Supporting Information: ctr70607‐supp‐0003‐Table S3.docx [file CTR-40-e70607-s001.docx]

**Table S3: Length of Stay among SPT recipients**

|  | Correlation Coefficient (95% CI) |
| --- | --- |
|  |  |
| Any component positive | 0.06 (-0.10 to 0.23; p= 0.45) |
| Reduced functionality | -0.01 (-0.18 to 0.15; p= 0.91) |
