# Supplementary material for: Pretransplant Malnutrition Risk Components are Associated With Adverse Outcomes After Simultaneous Pancreas and Kidney and Solitary Pancreas Transplantation
Source: Clin Transplant. 2026 Jul 2;40(7):e70607. doi: 10.1111/ctr.70607 (PMC13325689; doi:10.1111/ctr.70607)
Supplement: Supplementary file 4 — Supporting Information: ctr70607‐supp‐0004‐Table S4.docx [file CTR-40-e70607-s003.docx]

**Table S4: Comparison of outcomes of interest among SPT recipients**

| Outcomes of interest | At least one positive component | None of the positive components | p |
| --- | --- | --- | --- |
| Early readmission | 7 (54) | 46 (37) | 0.25 |
| Cardiovascular events | 1 (8) | 6 (5) | 0.66 |
| Pancreas acute rejection | 2 (15) | 34 (28) | 0.34 |
| Pancreas uncensored graft failure | 5 (39) | 32 (26) | 0.34 |
| Pancreas death censored graft failure | 5 (39) | 25 (20) | 0.13 |
| Death with functioning graft | 0 | 7 (6) | 0.38 |
